# Application of hybridization control probe to increase accuracy on ligation detection or minisequencing diagnostic microarrays

**DOI:** 10.1186/1756-0500-2-249

**Published:** 2009-12-14

**Authors:** Jarmo Ritari, Lars Paulin, Jenni Hultman, Petri Auvinen

**Affiliations:** 1 DNA sequencing and genomics laboratory, Institute of Biotechnology, University of Helsinki, 00790 Helsinki, Finland

## Abstract

**Background:**

Nucleic acid detection based on ligation reaction or single nucleotide extension of ssDNA probes followed by tag microarray hybridization provides an accurate and sensitive detection tool for various diagnostic purposes. Since microarray quality is crucial for reliable detection, these methods can benefit from correcting for microarray artefacts using specifically adapted techniques.

**Findings:**

Here we demonstrate the application of a per-spot hybridization control oligonucleotide probe and a novel way of computing normalization for tag array data. The method takes into account the absolute value of the detection probe signal and the variability in the control probe signal to significantly alleviate problems caused by artefacts and noise on low quality microarrays.

**Conclusions:**

Diagnostic microarray platforms require experimental and computational tools to enable efficient correction of array artefacts. The techniques presented here improve the signal to noise ratio and help in determining true positives with better statistical significance and in allowing the use of arrays with poor quality that would otherwise be discarded.

## Background

**Figure 1:**
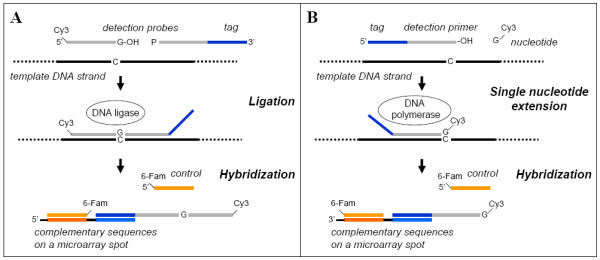
**Principle of ligation detection and minisequencing reactions**. A) Two ssDNA probes with one carrying a 5' fluorescent label and the other having a 5' phosphorylation and a 3' flanking tag sequence hybridize adjacently on target DNA. DNA ligase accepts nicked dsDNA strand as a substrate and joins the probes together if there is a perfect match to the template at the 3' end of the probe. B) Minisequencing is a similar approach using a detection primer and single dideoxynucleotide extension catalysed by DNA polymerase. The tag is in the 5' end of the primer and the nucleotide carries a fluorescent label incorporated into the 3' end. The reaction products are hybridized onto a microarray with complementary tag sequences. Each spot also harbors a complementary sequence to a 5' fluorescently labeled control probe which is read on another wavelength channel.

Nucleic acid detection by ligation and single-nucleotide extension minisequencing techniques take advantage of the catalytic selectivity of DNA ligase and polymerase enzymes, respectively, to recognize a unique position in a target DNA strand. In ligation assays, two specific ssDNA oligonucleotide detection probes are designed to hybridize adjacently on target DNA strand so that the 3' end of the label-carrying probe recognizes a discriminating position and is ligated to the phosphorylated 5' end of the other probe in the presence of a matching target molecule (figure 1A) [1,2]. Ligation detection can also be implemented as a single probe which is circularized upon ligation [3]. In minisequencing, the target is recognized through the addition of a specific labeled dideoxynucleotide to the 3' end of the oligonucleotide detection primer annealed immediately upstream of a discriminating position in the target (figure 1B) [4,5]. Both methods allow tagging of the probes for detection on a microarray platform containing complementary tag sequences providing uniform thermodynamic hybridization properties for all probes. The relatively high throughput and superior accuracy over traditional microarray and PCR based methods have motivated the application of ligation and minisequencing probe microarrays to SNP [6,7] and gene variant detection [8], clinical microbial diagnostics [9-11] and more recently to environmental microbiology [12-14]

Even though enzyme aided recognition provides good accuracy as such, ultimately sensitivity and reliability are dependent on microarray quality and successful hybridization. The fidelity of recorded intensities of hybridized detection probes might be adequate for diagnostics provided the array spot quality is constantly good throughout the array as is often the case with *in situ *synthesized and other high quality microarrays. However, aberrant spots that vary in morphology or DNA content can occur for instance in contact printed arrays, causing problems with accuracy of spot finding and quantification. In addition, the printing process can introduce additional background noise impeding the read-out of results. Therefore, diagnostic microarrays may benefit from information processing steps to remove biases and noise, but this requires additional experimental measures to determine the source of variance. Methods used in gene expression normalization are not directly applicable because they typically rely on the bulk signal of all array spots assuming only that a small minority of genes are differentially expressed (reviewed in [15,16]). In diagnostic microarrays, this assumption does not generally hold and also in some applications the number of spots per array is much lower.

## Method

**Figure 2:**
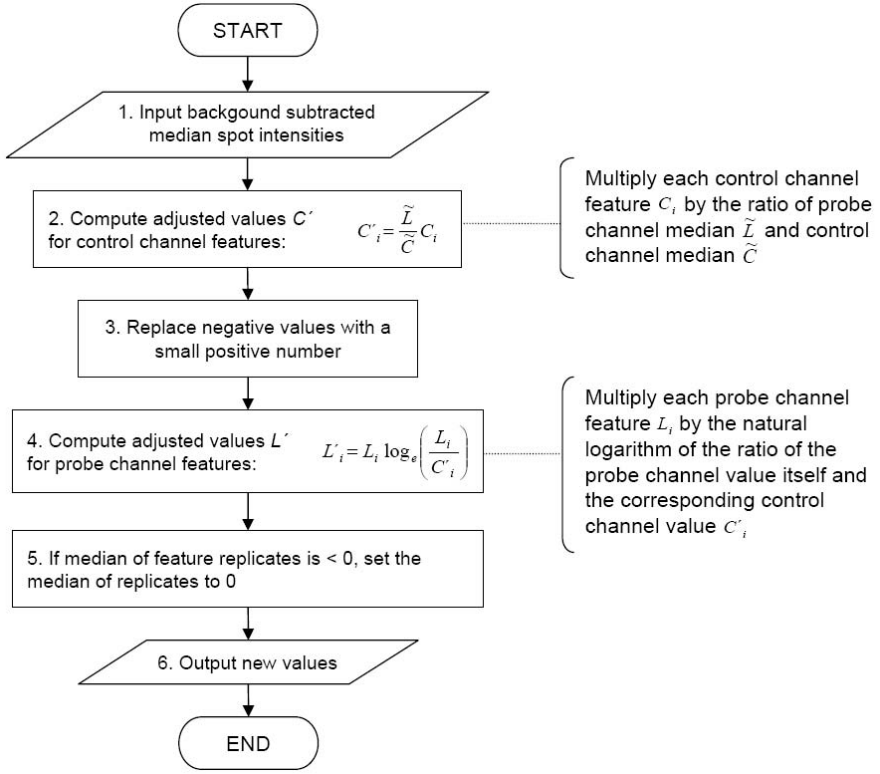
**Flow diagram describing computational steps of the normalization algorithm**. Input data are median background subtracted median spot pixel intensities. In the second step, control channel spot intensities are adjusted to the level of probe channel intensities. Next, negative values are replaced by a small number to avoid taking negative logarithm. Fourth, probe channel spot values are adjusted by multiplying the probe spot value by natural logarithm of the corresponding ratio of probe and control spot values. Finally, if median of replicate spots is negative, the difference is added to the spot values to set the median to the minimum of zero.

**Figure 3:**
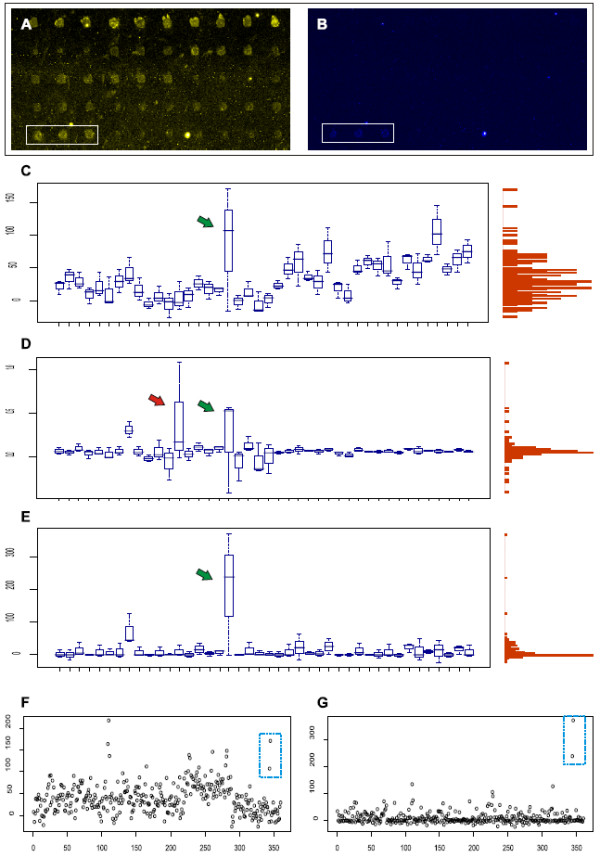
**An example of a microarray with high background noise and poor spot quality**. Plots showing results before and after applying the normalization procedure. A) and B) show a part of a microarray scanned in two channels; wavelength 488 (control with 6-Fam dye) and wavelength 532 (probes with Cy3 dye), respectively. The marked spot triplet in the bottom left corner shows a positive probe. Boxplots in C) show hybridization results of 42 detection probes with a positive probe indicated by green arrow. The probes are listed on the x-axis and their relative intensities on the right y-axis. On the left y-axis, a vertical histogram depicting the intensity distribution. The values are background subtracted detection probe channel signal intensities. D) shows the ratios of detection probe to adjusted control. Red arrow points a false positive. In E), the normalization described here is applied to the data, making positive signals better defined. F) and G) show all spots in the array before and after normalization, respectively. Marked spots in the top right corner indicate the positive probe.

Here we report an approach using a hybridization control oligonucleotide probe to measure tag array spot quality independently of detection probes (figure 1) and to enable normalization in order to remove noise and standardize signals between spots and subarrays (figure 2). The control probe positive for all spots is similar to the tag oligos with regard to length and base composition. The detection probe signal intensities are compared to the control probe signal intensities for each spot to obtain normalized signals. However, simply dividing the probe signals by control can be problematic if a spot has reduced intensity as a result of abnormal morphology or otherwise compromised quality. These kind of features can give rise to false positives when the probe channel is empty (due to no target present in the sample) because the background signal in the probe channel is relatively constant and independent of the quality of the spot. The control signal, on the other hand, reflects the spot quality much more closely because the control probe is positive to all spots. Thus, division of low but constant detection probe background signal by reduced control signal may produce artificially high ratio values (an example is given in figure 3).

To avoid generating false positives in the analysis, we have first computationally adjusted the control channel intensities to the level of detection probe channel median of all spots on a subarray in order to prevent the typically stronger control probe signal from dominating over the detection probe signal. The assumption behind this is that the median value represents empty detection probe signal. Next, the probe to control ratios are computed and used in logarithm as weighting coefficients in computing adjusted values for detection probe spot signals (figure 2). The advantage of this approach is that the probe signal value is multiplied by the weighting coefficient value over zero only if the detection probe signal is stronger than the control in that particular spot. If the spot is empty in the detection probe channel, the result of multiplication by the log ratio gives a small number even if the log ratio would be positive, unlikely to cause any false calls. In addition, as the log ratio and consequently the output is 0 with equal probe and control values, it provides a common reference point for all spots within and between microarrays.

## Implementation

The probe set used in the normalization experiments consisted of ligation detection probes similar to a previous study [13]. Forty-two different probes (the sequences are to be published elsewhere) were multiplexed in ligation reactions. The ligation reactions and hybridization conditions were as described previously [13]. Briefly, the ligations were carried out in a final volume of 20 μl containing 1× ligation buffer (TAQ ligase buffer, New England Biolabs, MA, USA), 30 mM tetramethylammonium chloride (TMAC), 250 fmol of each discriminating probe, 250 fmol of each common probe, 5 pmol of the complementary hybridisation control probe, a variable amount of purified PCR products and 4 U of Taq DNA ligase (New England Biolabs). The reaction was cycled for 40 rounds at 94°C for 30 s and at 60-64°C for 4 min in a thermocycler (MJ Research). The LDR mix (20 μl) was diluted to obtain 40 μl of hybridization mixture containing 5× SSC and 0.1 mg/ml herring sperm DNA. After heating the mix to 94°C for 2 min and chilling on ice, ligation control probe was added and the mix was applied onto the slide. The microarray slides were produced by contact printing by Telechem (CA, USA) or by university core facility (Biomedicum Biochip Center, University of Helsinki, Finland). The microarrays had 16 subarrays each, consisting of 119 tag oligos in triplicates [see Additional file 1]. Scanning were done as described previously [13]. All computations were done in R-software environment (2.8.0) [17], using the Bioconductor package Marray (1.20.0) [18] [see Additional file 2] for reading GenePix result (gpr) files [see Additional file 3]. In order to evaluate the effect of normalization, no filtering or outlier removal procedures were applied to the data.

## Results

The normalization procedure was tested with two different sets of detection probes on microarrays containing either three or five spot replicates. Some subarrays had printing artefacts like background noise or low quality spots. Figure 3 shows an example of a typical poor quality subarray. In these kind of arrays, the spot morphology varied considerably and background noise was high in places resulting in signal-to-noise ratio much lower than in regular microarrays. Comparison of results before and after applying the control probe and normalization demonstrates the impact of these procedures on the signal-to-noise ratio. Clearly, just subtracting background signal from each spot raw value in the detection probe channel is not enough to provide reliable results (figure 3C). Computing signal ratio of the detection probe to the adjusted control (figure 3D) greatly reduces variation in the background distribution but at the same time weakens true positive signal and causes some spots to falsely reach too high values. The normalization procedure presented here, however, avoids these pitfalls and is able to correct for the variation and keep the true positive signal clear (figure 3E and 3G). We tested 8 microarray slides with altogether 128 subarrays. Furthermore, the procedure was applied on data from previously published microarray hybridizations [see Additional file 4]. In this set, the ligation probes were designed against environmental fungi [13] and detected on a tag microarray with five spot replicates. Also in this case the normalization was capable of correcting microarray noise. As expected, the computations had little or no effect on results from good quality microarrays [see Additional file 5].

## Discussion

DNA microarrays, while being a potential tool for diagnostics, typically have irregularities in spot quality causing problems with accuracy of detection. Efficient background correction is required in ligation, minisequencing and similar diagnostic systems where standard gene expression analysis methods may not apply. Although high quality microarrays are preferable for diagnostics, they may not always be economically feasible for routine use due to high costs. The prices of customizable commercial high-density *in situ *synthesized microarrays are likely decrease but these kind of platforms can still benefit from noise correction as human error in performing the hybridizations can not be ruled out. In addition, some emerging diagnostic platforms such as integrated microfluidic systems can take advantage of microarrays for detection of probes on a small scale [19,20]. The mass fabrication process of the devices and hybridization conditions might bring about variation in spot quality and available space for replicates in microsystems is likely to be limited as well. In diagnostic applications in general, accuracy of the results is highly important and proper correction procedures can help deal with noise to increase statistical reliability, making the system practicable even if the detection platform is not fully optimal.

We found the normalization procedure based on an internal control for each assay spot to be useful when working with tag microarrays having compromised spot quality and background noise. A similar approach has been used by others before to monitor array spot quality and to correct for printing variations. In a study by Ye and coworkers, amplicons were first generated by forward and reverse primers carrying different labels [21]. One strand of a amplicon served as target for SNP recognition probes while the other strand was used as a internal control hybridizing to a complementary control probe in the same spot. In another work, a 25 mer control oligo was spotted alongside with 70 mer recognition probes to monitor spot quality with a complementary labeled 25 mer [22]. In both of these studies, a given spot control signal was first compared with the mean control signal and the obtained value was then used to divide the detection signal of the same spot. Similarly, Akhras and coworkers computed signal ratios of detection probe vs. an all-positive control on a padlock probe tag array [9]. However, problematic microarrays or abnormal spots were not the focus of these studies and correcting microarray defects was not discussed in further detail. The method presented here uses a similar principle of internal control but different computational procedure to effectively reduce noise on low quality spots, emphasizing signal extraction rather than mere elimination of problematic spots. It is important not only to monitor the array spot quality but also to take into account the possible bias in probe to control ratio to effectively process aberrant spots on low quality arrays.

We have also demonstrated earlier with cDNA microarrays that effective measurement of spot quality with an additional dye improves the reliability of detection [23]. Using an all-positive control probe serves a similar idea in assisting spot quantification which is highly dependent on the accurate determination of the true spot area to estimate spot and background signal intensities. This is especially relevant if the signal intensity of a spot is low in the detection channel. The control channel, having a relatively high intensity in all spots, helps in locating the spots and capturing their areas more accurately. However, it should be noted that our method does not use all the information available in the control channel. For instance, the control signal could be used to model the detection probe channel intensity profile. This approach could potentially increase the signal-to-noise-ratio of the detection probe channel on weak spots, opening the possibility for further development of the method.

The computation presented here can be easily implemented in any freely programmable system used for microarray data analysis. It should be noted however, that application of the control probe requires that each spot on the tag microarray harbors the complementary control sequence, along with the actual tag sequence. One way to overcome the need to synthesize novel long probes is to mix control oligonucleotide serving as an internal control with each of the speficic oligonucleotides and deposit these mixture on arrays [22]. In addition, if it is expected that over 50% of detection probes should be positive in an experiment, negative control spots are needed to compute the normalization. This is because the procedure assumes that the detection probe channel median represents empty signal in computing adjusted signal values for the control channel.

## Conclusions

Much of the noise introduced by variable spot quality on detection probe read-outs can be corrected by applying simple computations. This involves adjusting the control probe signals to detection probe signal levels and taking into account absolute value of the detection probe signal and variability in the control probe signal. The method is potentially advantageous in various diagnostic microarray platforms.

## Competing interests

The authors declare that they have no competing interests.

## Authors' contributions

JR analysed the data and wrote the scripts. JR, LP, JH and PA conceived of the study and drafted the manuscript. All authors read and approved the final manuscript.

## Supplementary Material

Additional file 1 **Tag_sequences**. Sequences of tags printed on microarrays and complementary tags flanking the detection probes. Click here for file

Additional file 2 **Scripts**. R file containing scripts for reading data, computing normalization and drawing boxplots. Click here for file

Additional file 3 **Gpr_files**. Zip-compressed file containing raw microarray data in GenePix results format (.gpr) and GAL (GenePix Array List) files. Click here for file

Additional file 4 **Supplemental_figure1**. Boxplots showing data from a different probeset published previously [13]. The probes are listed on the x-axis and their relative intensities on the right y-axis. On the left y-axis, a vertical histogram depicting the intensity distribution. Signals before (A) and after (B) the normalization procedure. The probes are designed to detect different environmental fungi and were hybridezed on microarray with tag sequences spotted in 5 replicates. The normalization is capable of correcting noise and making the signal from true positive *Penicillium commune *probe (tag A50) clearer as indicated by blue arrows. For the sake of clarity, another positive probe on tag A29 was left out from the plot. Tags A100 and A101 represent empty spots (i.e. without corresponding detection probes). Click here for file

Additional file 5 **Supplemental_figure2**. Boxplots showing data from a good quality microarray before (A) and after (B) normalization. The probes are listed on the x-axis and their relative intensities on the right y-axis. On the left y-axis, a vertical histogram depicting the intensity distribution. Click here for file
