# Supplementary material for: Application of hybridization control probe to increase accuracy on ligation detection or minisequencing diagnostic microarrays
Source: BMC Res Notes. 2009 Dec 14;2:249. doi: 10.1186/1756-0500-2-249 (PMC2799435; doi:10.1186/1756-0500-2-249)
Supplement: Additional file 2 — Scripts. R file containing scripts for reading data, computing normalization and drawing boxplots. [file 1756-0500-2-249-S2.RTF]

##
## Functions for reading GenePix result files
## and computing normalization and boxplots.
##
## contact: jarmo.ritari@helsinki.fi
##


library(marray)

## function for data read-in
read.gpr <- function(gprs, annotation, label_info) { ## .gpr, .gal, subarraynames
	datas <- list()
	anno <- read.Galfile(annotation)
	for(i in 1:length(gprs)) {
		read.GenePix(fnames=gprs[i],
			name.Gf="F532 Median - B532",		
			name.Gb="B532 Median", 
			name.Rf="F488 Median - B488",
			name.Rb="B488 Median" ) -> data
		anno$gnames[1:360] -> data@maGnames ## 3 x 120 = 360 spots
		label_info[i] -> data@maNotes
		data -> datas[i] 
	}
	datas
}


## function for normalization and plotting
box.plot.norm <- function(probes, datas, y.lim=NULL) {
	
	probe.list <- matrix(nrow=3, ncol=length(probes))
	## get values for probes in the list
	for ( a in seq(length(probes)) ) {
		which(as.character(datas@maGnames@maLabels) %in% probes[a]) -> probe.list[,a] 
	}
		
	Rf.new	<- datas@maRf[1:360]	## control channel		
	medR		<- median(datas@maRf[1:360])	
	medG		<- median(datas@maGf[1:360])
	med.ratio	<- medG/medR

	Rf.new	<- datas@maRf[1:360]*med.ratio
	Rf.new	<- mapply(Rf.new, FUN=remove.neg)
		
	Gf.new  	<- datas@maGf[1:360]  	## detection probe channel
	Gf.new	<- mapply(Gf.new, FUN=remove.neg)
		
	probesG 		<- Gf.new[as.vector(probe.list)]
	probesR 		<- Rf.new[as.vector(probe.list)]	
	label.factor 	<- factor(rep(probes, each=3), levels=probes, ordered=T)
			
	probes.norm		<- probesG*(log(probesG/probesR))
	probes.norm[is.nan(probes.norm)] <- 0.01

	probes.norm	<- as.vector(unlist(tapply(probes.norm, INDEX=label.factor, 
				FUN=level, reference.median=median(probes.norm))))
				## the input to reference.median should represent 
				## negative features
				
	windows()

	## values for vertical histogram
	def.par 	<- par(no.readonly = TRUE)  
	yhist 	<- hist(probes.norm, breaks=100, plot=FALSE) 
	top 		<- max(yhist$counts)
	yrange 	<- c(min(probes.norm), max(probes.norm))
	nf 		<- layout(matrix(c(1,2,1,2),2,2,byrow=T), c(5,1), c(1,5), F)
	layout.show(nf)

	par(mar=c(4,3,2,1), las=3)
	## drawing the boxplots 
	boxplot((probes.norm)~label.factor, main=datas@maNotes, 
		border=c('darkblue'), ylim=y.lim)
	par(mar=c(4,0,2,1)) 
	## drawing the histogram
	barplot(yhist$counts, axes=F, xlim=c(0, top),  
		space=0, horiz=TRUE, col='orangered3',border=NA, xpd=F) 
	
	par(def.par) 
	
}	


##
## additional functions required by box.plot.norm()
##

level <- function(x, reference.median) {
	reference.median - median(x) -> diff
	if(diff > 0) {
		x <- x + diff	
	}
	x
}
	
remove.neg <- function(x) {	
	if(x < 0){
		x <- 0.1
	} 
	x
}


##
## usage
##

## the tag names of the probes in the set
probes <- c('99',  '100', '101', '102', '103', '104',
		'105', '106', '107', '108', '109', '110',
		'111', '112', '113', '114', '115', '116',
		'117', '118', '119', '120', '29',  '52',
		'53',  '54', '55',  '56',  '57',  '58',
		'59',  '83',  '84',  '85',  '25',  '14',
		'86',  '87',  '88',  '89',  '90',  '91')
paste('A', probes, sep='') -> probes


## .gpr files in the current working dir.
## this example lists Slide 1 subarrays
results <-c("lasi1_1-16.gpr", "lasi1_2-16.gpr",   
		"lasi1_3-16.gpr", "lasi1_4-16.gpr", 
		"lasi1_5-16.gpr", "lasi1_6-16.gpr",   
		"lasi1_7-16.gpr", "lasi1_8-16.gpr",
		"lasi1_9-16.gpr", "lasi1_10-16.gpr", 
		"lasi1_11-16.gpr","lasi1_12-16.gpr", 
		"lasi1_13-16.gpr","lasi1_14-16.gpr", 
		"lasi1_15-16.gpr","lasi1_16-16.gpr")           		

## to list .gpr files in the working dir:
## list.files(pattern='.gpr') 


## label for each .gpr file
## order must be same as in 'results'
labels <-  c("Slide1 B1", "Slide1 A1",   
		"Slide1 B2", "Slide1 A2",
		"Slide1 B3", "Slide1 A3", 
		"Slide1 B4", "Slide1 A4",
		"Slide1 B5", "Slide1 A5",
		"Slide1 B6", "Slide1 A6",
		"Slide1 B7", "Slide1 A7",
		"Slide1 B8", "Slide1 A8")        
   		
## to generate names for each subarray on a slide:
#labels <- paste('Slide1', rep(c('B','A'), times=16), rep(1:8, each=2))


## draw boxplots of all 16 subarrays in separate windows: 
lapply(FUN=box.plot.norm, data, probes=probes, y.lim=NULL)

## draw a boxplot of a single subarray (B8 in this example): 
box.plot.norm(probes=probes, data[[16]], y.lim=NULL)
