# Supplementary figures and images for: Application of hybridization control probe to increase accuracy on ligation detection or minisequencing diagnostic microarrays
Source: BMC Res Notes. 2009 Dec 14;2:249. doi: 10.1186/1756-0500-2-249 (PMC2799435; doi:10.1186/1756-0500-2-249)

A

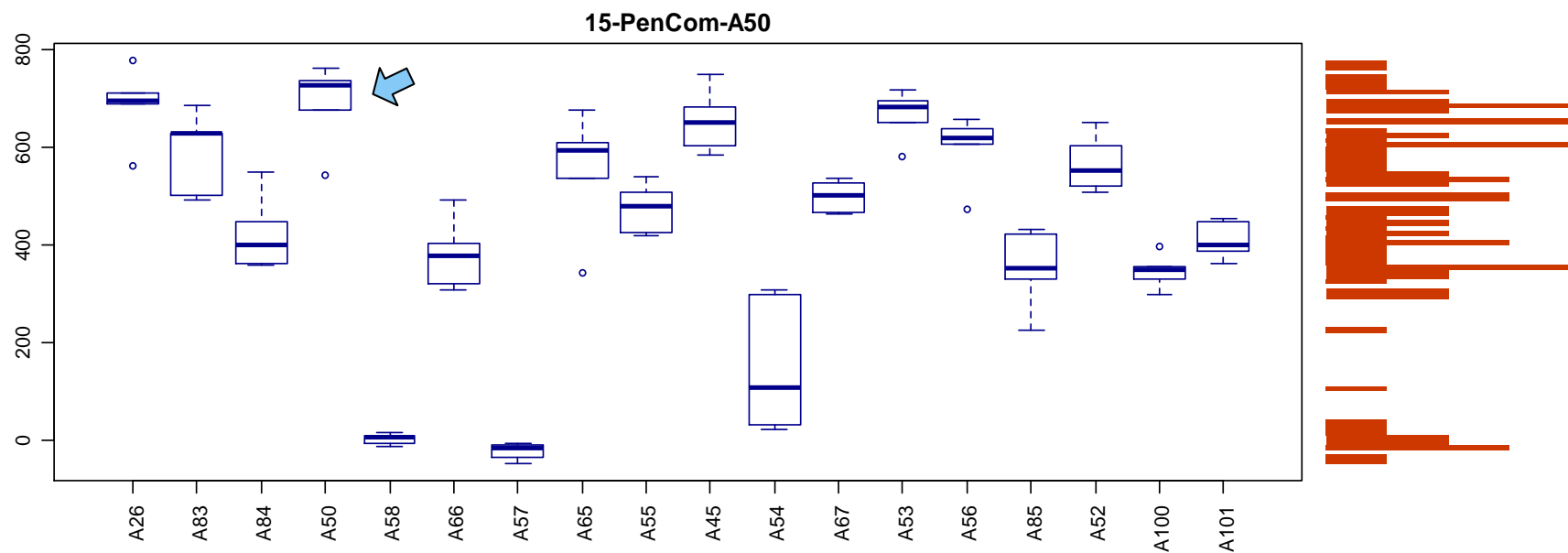

B

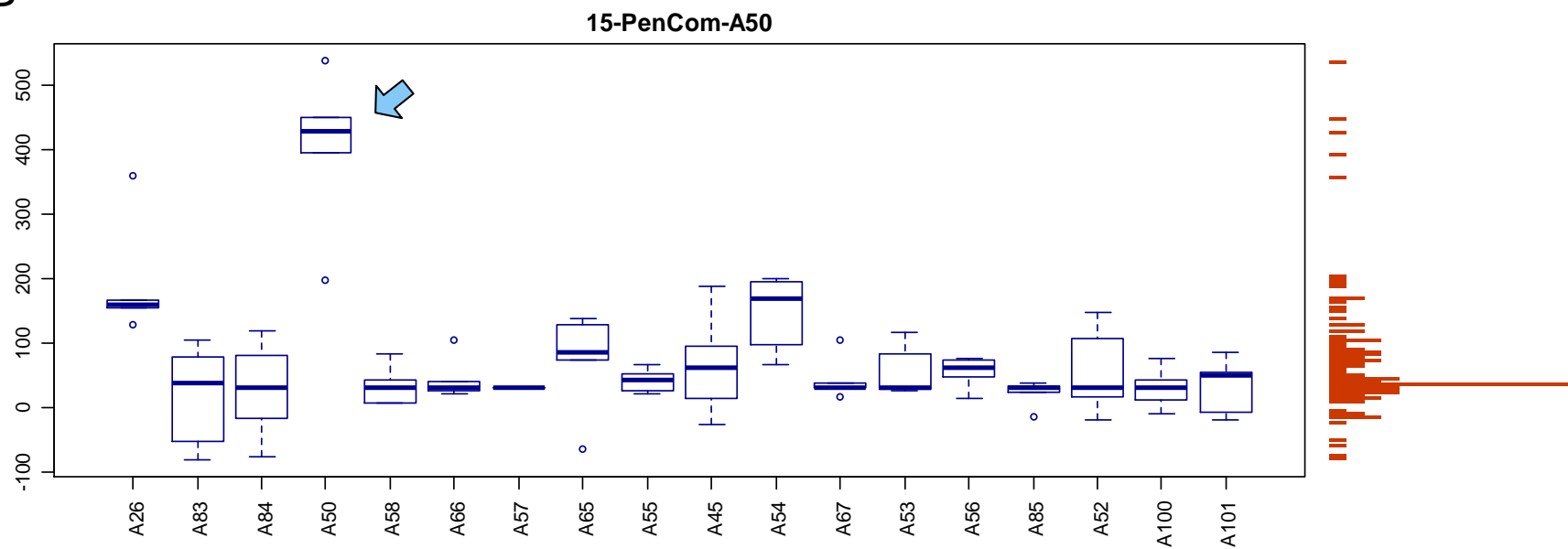

Supplement: Additional file 4 — Supplemental_figure1. Boxplots showing data from a different probeset published previously [13]. The probes are listed on the x-axis and their relative intensities on the right y-axis. On the left y-axis, a vertical histogram depicting the intensity distribution. Signals before (A) and after (B) the normalization procedure. The probes are designed to detect different environmental fungi and were hybridezed on microarray with tag sequences spotted in 5 replicates. The normalization is capable of correcting noise and making the signal from true positive Penicillium commune probe (tag A50) clearer as indicated by blue arrows. For the sake of clarity, another positive probe on tag A29 was left out from the plot. Tags A100 and A101 represent empty spots (i.e. without corresponding detection probes). [file 1756-0500-2-249-S4.PDF]

A

14.Panfungal-A29

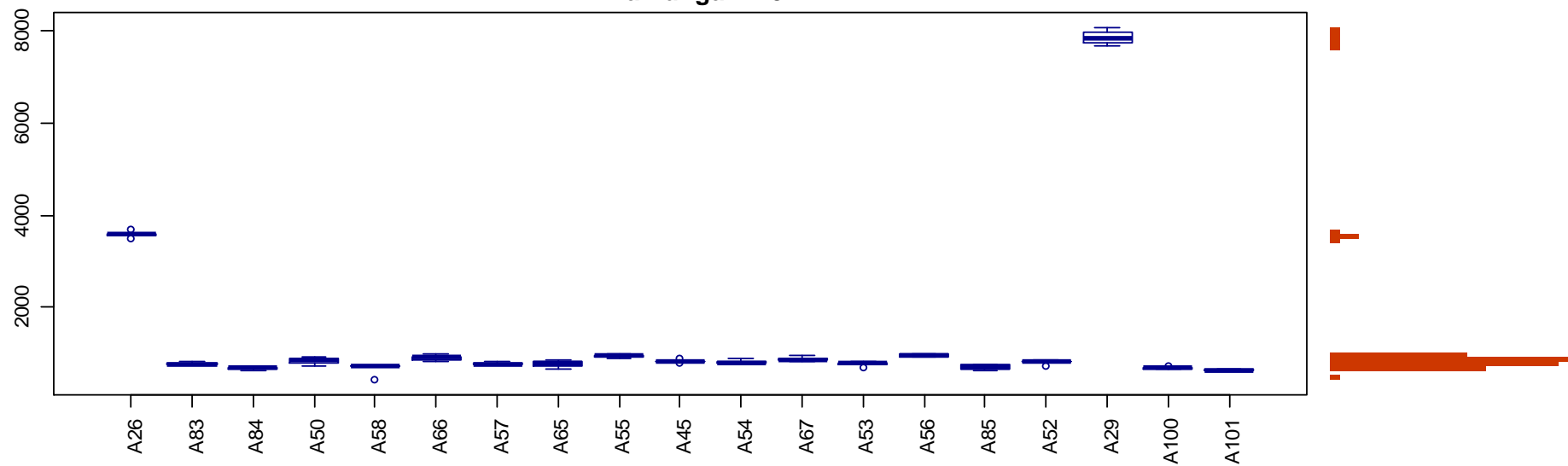

B

14.Panfungal-A29

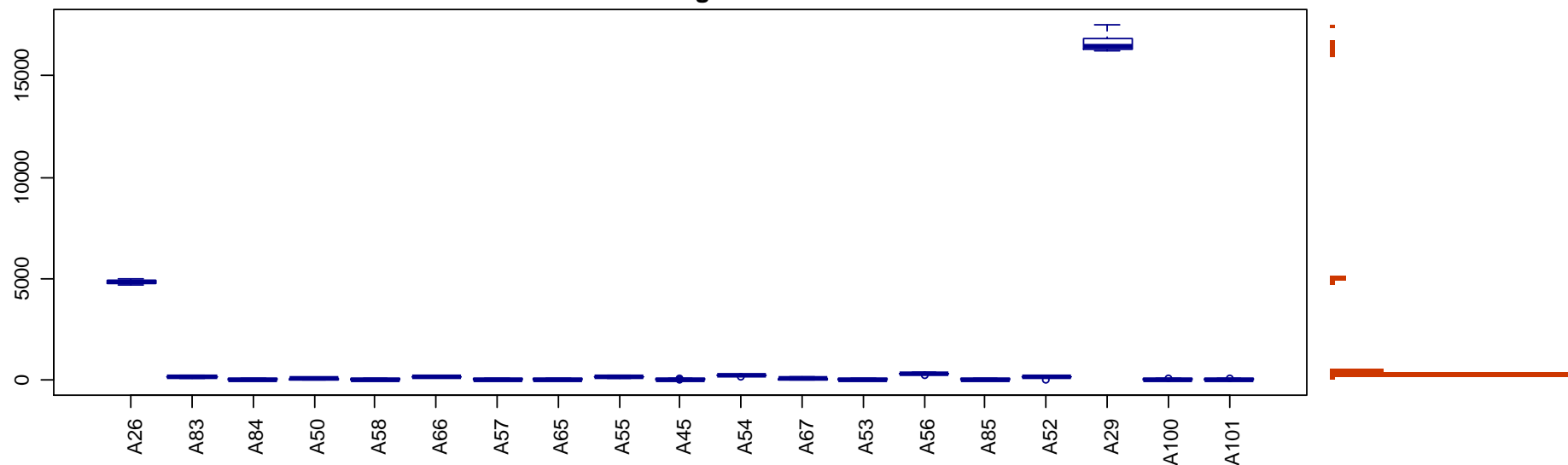

Supplement: Additional file 5 — Supplemental_figure2. Boxplots showing data from a good quality microarray before (A) and after (B) normalization. The probes are listed on the x-axis and their relative intensities on the right y-axis. On the left y-axis, a vertical histogram depicting the intensity distribution. [file 1756-0500-2-249-S5.PDF]
